# The Association Between Prenatal Exposure to Antidepressants and Autism: Some Research and Public Health Aspects

**DOI:** 10.3389/fpsyt.2020.555740

**Published:** 2020-11-23

**Authors:** Ori Kapra, Ran Rotem, Raz Gross

**Affiliations:** ^1^Department of Epidemiology and Preventive Medicine, Sackler Faculty of Medicine, School of Public Health, Tel Aviv University, Tel-Aviv, Israel; ^2^School of Public Health, Harvard University, Boston, MA, United States; ^3^Morris Kahn Maccabi Health Data Science Institute, Tel-Aviv, Israel; ^4^Sheba Medical Center, Ramat Gan, Israel; ^5^Department of Psychiatry, Sackler Faculty of Medicine, Sackler School of Medicine, Tel Aviv University, Tel Aviv, Israel

**Keywords:** autism (ASD), antidepressant (AD), SSRI (selective serotonin reuptake inhibitor), prenatal, maternal depression, confounding by indication, *in utero* development

## Abstract

Use of antidepressants (ADs) in general, and in pregnant notwithstanding, has been increasing globally in recent decades. Associations with a wide range of adverse perinatal and childhood outcomes following prenatal ADs exposure have been observed in registry-based studies, with Autism Spectrum Disorders (ASD) frequently reported. Studies using animal models, sibling analyses, and negative control approaches, have linked dysfunctional serotonin metabolism with ASD, but did not convincingly tease apart the role of maternal mental health from that of ADs. As work to decipher the nature of the AD-ASD association continues, this review raises some public health concerns pertinent to a hypothetical conclusion that this association is causal, including the need to identify specific gestation periods with higher risk, the importance of precise assessment of the ASD potential prevention that might be attributed to AD discontinuation, and the estimation of risks associated with prenatal exposure to untreated depression.

## Introduction

Use of antidepressants (ADs) in pregnancy results in fetal exposure to the medications, in concentrations that are 70–80% of maternal serum levels (1). Yet, fetal exposure to ADs has not received the same clinical, ethical, or sociological attention that ADs use in children and adolescents has. From an ethical and public health research perspective, this topic introduces some key issues. First, unlike children and adolescents who are generally treated following the medical principle of *primum non nocere* (“First do not harm”), i.e., treating only those who have demonstrated sufficient impairment such that the benefits of treatment outweigh potential harm by adverse effects, fetuses are “innocent bystanders,” passively exposed to AD. This is evident in studies suggesting the use of selective serotonin reuptake inhibitors (SSRIs), a class of AD, has been increasing globally for the last 20–30 years, particularly among women and during pregnancy (2–8). Second, the choices facing physicians and pregnant women who struggle with mental health problems are complex, since in a dissimilarity to other exposures such as tobacco or illicit drugs where the goal is to eliminate use in pregnancy *entirely*, discontinuing the use of SSRIs and leaving maternal depression untreated may by itself have deleterious consequences for both mother and child (9–11). Hence, somewhat unique to investigations of prenatal exposure to AD, and SSRIs in particular, is an epidemiological equivocality on whether one should look for the Number Needed to Harm (NNH) or the Number Needed to Treat (NNT). Over the last decade, this conceptual ambiguity has not deterred the establishing of a consistent association with another public health concern, namely autism spectrum disorder (ASD) (12, 13).

## The Association Between Prenatal SSRIs and Autism Spectrum Disorder (ASD)

Twin studies have proposed that up to 70% of monozygotic pairs are concordant for autism, while up to ~90% are concordant for a broader spectrum of related cognitive or social abnormalities (14, 15). These heritability figures suggest that ASD is largely genetic, while providing substantial evidence that non-heritable factors are also likely to have an etiological role (16). Frequently mentioned among such prenatal exposures is use of SSRIs medications.

Fetal exposure to SSRIs is suspected as having significant consequences, mainly because well-before it assumes its renowned role as a neurotransmitter in adulthood, serotonin (5-HT) serves as a neural growth factor in gestation, critically influencing the brain structure and function of the developing fetus (17–22). All SSRIs cross the placenta, and while the mechanism by which SSRIs can potentially become hazardous during gestation is not entirely clear, associations with a wide range of adverse perinatal problems have been observed, including reduced fetal head growth (23), low birth weight (24), neural tube defects (25, 26), cardiac malformations (27), specifically atrial and ventricular defects (28), craniosynostosis (28), persistent pulmonary hypertension (29, 30), and lower APGAR scores (31).

**Figure 1:**
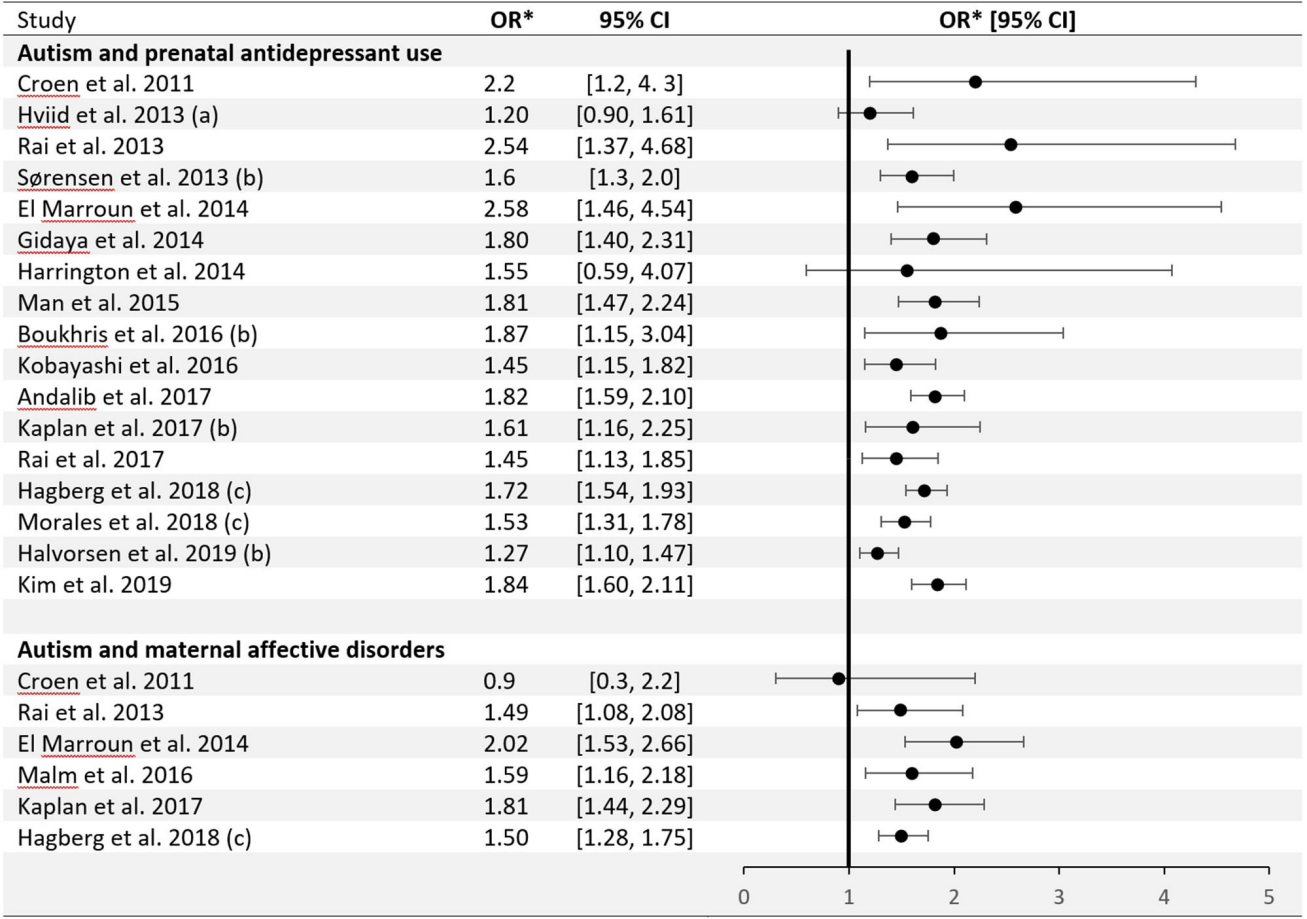
Epidemiological studies of the association between autism and prenatal antidepressant use/maternal affective disorders: odds ratio (± 95% Confidence Interval). *Eight individual studies reported other outcome measures: **(a)** Rate Ratio; **(b)** Hazard Ratio; **(c)** Relative Risk.

Among childhood neuropsychiatric disorders, ASD has been associated with prenatal SSRIs exposure (32). Figure 1 shows that over the last decade observational studies have formed an aggregate of positive findings, with little, if any variety in their effect estimates (33–49). One meta analysis suggested a pooled odds ratio of 1.82, with a 95% Confidence Interval (CI) = 1.59–2.10 (32) when comparing exposed and unexposed fetuses, or an adjusted odds ratio of 1.81 (95% CI = 1.47–2.24) with non-significant heterogeneity of the effect estimates across studies (respectively: *Q* = 3.61, *P* = 0.73; *Q* = 0.5, *P* = 0.92) (45). Only a single meta-analysis to date produced null findings for the ASD-SSRI association (adjusted RR = 1.13, 95% CI = 0.93–1.39) (50). This null finding has been interpreted by the authors as a failure of other meta-analyses to account for publication bias. The latest systematic review and meta-analysis comprising of 20 studies had calculated positive pooled Hazard Ratios (HR) for both cohort and case-control designs (respectively: HR = 1.27, 95% CI = 1.10–1.47; HR = 1.60, 95% CI = 1.26–2.02), and similarly drew attention that a publication bias cannot be precluded from influencing the results (40). Still, the observed association between SSRIs and developmental disorders is currently hardly in contention. The possible causality implied by it, however, is debatable.

Several investigative strategies to assess the causality of the association between maternal AD use during pregnancy and ASD risk in progeny have emerged. Results from studies using animal models have strengthened hypothesis that early SSRIs exposure may alter normal brain development, hippocampal neurogenesis, and epigenetic programming (51–55). In humans, epidemiological samples have been studied by means of diverse analytic approaches, each presenting unique challenges. Regression models adjusted for maternal mental illness are limited due to established underreporting of depression (30, 56–60), which leads to incomplete control for confounding by indication or by depression severity, with the latter potentially also confounding the dose-response gradient between SSRI exposure and risk of ASD. Confining the analysis only to children born to mothers with a history of psychiatric disorders indeed seems to attenuate or nullify the SSRI-ASD association (3), as observed in one systematic review (46) and one meta-analysis (39) (respectively: pooled adjusted risk ratio = 1.18, 9%% CI = 0.91–1.52; pooled odds ratio = 0.96, 95% CI = 0.57–1.6).

Figure 1 also shows studies demonstrating an association between maternal affective disorders and ASD, unconditioned on SSRIs use. The first and only meta-analysis to pool data on mothers with only psychiatric disorders but no SSRI exposure and those with SSRI exposure during pregnancy has shown elevated ASD risk in both groups (38). An additional method of accounting for confounding from genetic or environmental sources is discordant (on exposure) siblings analysis (61). It is important to acknowledge, however, that results of sibling-comparison studies may be biased if there are non-shared, time-varying confounders between siblings (62), as observed in one particular study (49). Negative control methods were utilized at two predominant levels: pre-conception use of SSRIs (36, 38) and paternal use of SSRIs (40, 63). Given the complicated use pattern of SSRIs and the variability of depressive states over time, these approaches may not be always valid (36).

In summary, of the empiric approaches to assessing the contribution of prenatal SSRIs exposure to ASD, none have been conclusively successful in teasing apart the role of maternal depression from that of medicinal treatment. The data are highly congruent, however, with one proposed biological mechanism of ASD development, serotonin metabolism dysfunctions, and serotonergic changes (64, 65). Along the described limitations in addressing indication bias, *in utero* SSRI exposure may potentially intervene with the end-point diagnosis as well (66). Mechanisms which impact adults' 5-HT can last after discontinuation of treatment, and can potentiality alter affective and cognitive function in infants and children painting a specific clinical picture without necessarily causing autism.

## Discussion

It is plausible that we may soon be able to answer the question “can prenatal exposure to SSRIs cause ASD?” with a greater degree of certainty, yet the causal nature of this association does not address this public health topic in its entirety. First, the potential risks of exposure to SSRIs have to be evaluated while also considering the potential effects of untreated maternal depression on the fetus. An altered intrauterine environment due to maternal depression-related effects on hypothalamo-pituitary-adrenal (HPA) activity has been linked with adverse neurodevelopmental outcomes (10). Second, even if a scientific consensus is reached that SSRIs *are* detrimental to the fetus, it is unlikely that medications carry a constant risk throughout the entire pregnancy, and critical gestation periods during which the fetus is potentially more vulnerable should be identified. Some studies associated elevated ASD risk with first trimester SSRIs exposure (34, 38, 67), while others linked adverse effects with SSRIs use in the second or third trimesters (45). Highlighting the above, a recent meta-analysis established that regardless of first trimester SSRI exposure, effects are also observed with second trimester exposure (40). Third, in the (currently hypothetical) case that a causal relationship is found, before considering amending the pregnancy safety category for SSRIs, it is recommended to have a sound estimation of the proportional ASD prevention that might be attributed to SSRI discontinuation, which as it currently stands appears to be very modest. It is estimated that between 0.6 and 2% of ASD cases in population-based cohort studies could have been prevented if prenatal SSRIs exposure were eliminated (36, 48). These estimations refute suggestions that the trend of increasing ASD prevalence is attributable in a meaningful way to the increased use of AD medications. The perception by the individual pregnant woman of the potential risk associated with *in-utero* exposures is known to be perceived as greater than it actually is (68). Yet balanced public health policy and clinical decision-making regarding AD use during pregnancy would likely lead to better health outcomes for both mother and offspring.

In conclusion, there is little contention that SSRIs are associated with ASD. No study to date, however, has ruled out the possibility of confounding or by other factors indication in a convincing way. As accumulating data on this topic support that serotonin metabolism may be in the causal pathway to ASD, there is need for novel approaches that could elucidate this conundrum (69).

## Author Contributions

OK and RG conceived the work and drafted the article. RR provided critical suggestions for revisions.

## Conflict of Interest

The authors declare that the research was conducted in the absence of any commercial or financial relationships that could be construed as a potential conflict of interest.
